# Confidence Interval Based Parameter Estimation—A New SOCR Applet and Activity

**DOI:** 10.1371/journal.pone.0019178

**Published:** 2011-05-31

**Authors:** Nicolas Christou, Ivo D. Dinov

**Affiliations:** 1 Department of Statistics, University of California Los Angeles, Los Angeles, California, United States of America; 2 Department of Statistics and Center for Computational Biology, University of California Los Angeles, Los Angeles, California, United States of America; Universidad Veracruzana, Mexico

## Abstract

Many scientific investigations depend on obtaining data-driven, accurate, robust and computationally-tractable parameter estimates. In the face of unavoidable intrinsic variability, there are different algorithmic approaches, prior assumptions and fundamental principles for computing point and interval estimates. Efficient and reliable parameter estimation is critical in making inference about observable experiments, summarizing process characteristics and prediction of experimental behaviors. In this manuscript, we demonstrate simulation, construction, validation and interpretation of confidence intervals, under various assumptions, using the interactive web-based tools provided by the Statistics Online Computational Resource (http://www.SOCR.ucla.edu). Specifically, we present confidence interval examples for population means, with known or unknown population standard deviation; population variance; population proportion (exact and approximate), as well as confidence intervals based on bootstrapping or the asymptotic properties of the maximum likelihood estimates. Like all SOCR resources, these confidence interval resources may be openly accessed via an Internet-connected Java-enabled browser. The SOCR confidence interval applet enables the user to empirically explore and investigate the effects of the confidence-level, the sample-size and parameter of interest on the corresponding confidence interval. Two applications of the new interval estimation computational library are presented. The first one is a simulation of confidence interval estimating the US unemployment rate and the second application demonstrates the computations of point and interval estimates of hippocampal surface complexity for Alzheimers disease patients, mild cognitive impairment subjects and asymptomatic controls.

## Introduction

### Variability and Estimation in Quantitative Studies

Solutions to many biological, engineering, social, environmental or health related challenges depend on obtaining accurate, robust and computationally-tractable parameter estimates. All natural processes, observable phenomena and designed experiments are affected by intrinsically or extrinsically induced variation [1]. Our understanding of such processes frequently revolves around estimating various population parameters of interest based on observed (acquired) data. Commonly used parameters of interest include measures of centrality (e.g., mean, modes), measures of variability (e.g., mean absolute deviation, range), measures of shape (e.g., skewness, kurtosis), proportions, quantiles, and many, many others. There are two types of parameter estimates - point-based and interval-based estimates. The former refer to unique quantitative estimates, and the latter represent ranges of plausible values for the parameters of interest. There are different algorithmic approaches, prior assumptions and principals for computing data-driven parameter estimates. These depend on the distribution of the process of interest, the available computational resources and other criteria that may be desirable [2], e.g., biasness and robustness of the estimates. Accurate, robust and efficient parameter estimation is critical in making inference about observable experiments, summarizing process characteristics and prediction of experimental behaviors.

**Figure 1:**
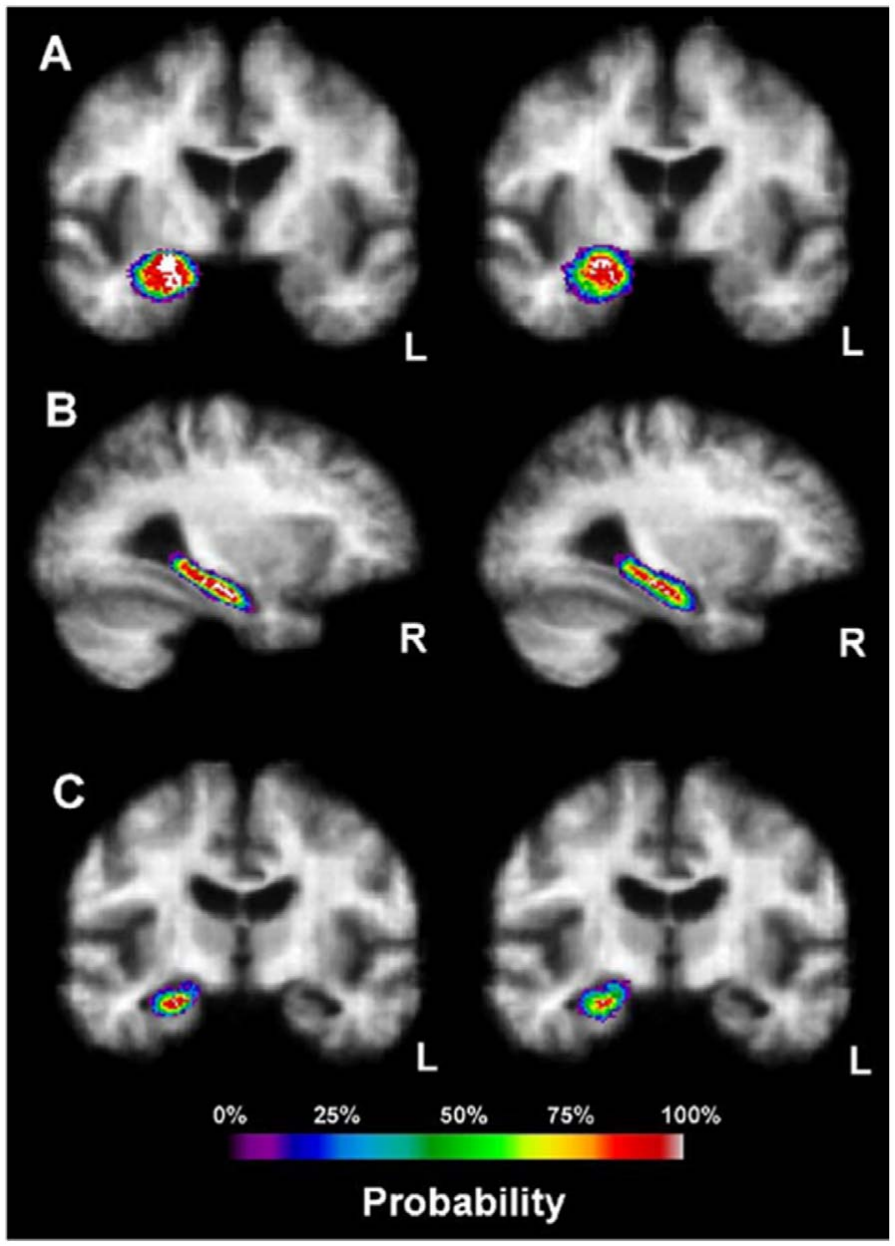
Biomedical example of interval-based parameter estimation - probabilistic representation of the 3D shape of the right hippocampus in the computational Alzheimer's disease brain atlas.

For example, a 2005 study proposing a new computational brain atlas for Alzheimer's disease [3] investigated the mean volumetric characteristics and the spectra of shapes and sizes of different cortical and subcortical brain regions for Alzheimer's patients, individuals with minor cognitive impairment and asymptomatic subjects. This study estimated several centrality and variability parameters for these populations. Based on these point- and interval-estimates, the study analyzed a number of digital scans to derive criteria for imaging-based classification of subjects based on the intensities of their 3D brain scans. Their results enabled a number of subsequent inference studies that quantified the effects of subject demographics (e.g., education level, familial history, APOE allele, etc.), stage of the disease and the efficacy of new drug treatments targeting Alzheimer's disease. Figure 1 illustrates the shape, center and distribution parameters for the 3D geometric structure of the right hippocampus in the AlzheimerÕs disease brain atlas. New imaging data can then be co-registered and quantitatively compared relative to the amount of anatomical variability encoded in this atlas. This enables automated, efficient and quantitative inference on large number of brain volumes. Examples of point and interval estimates computed in this atlas framework include the mean-intensity and mean shape location, and the standard deviation of intensities and the mean deviation of shape, respectively.

### The Statistics Online Computational Resource (SOCR)

The Statistics Online Computational Resource (SOCR) (http://www.socr.ucla.edu) is an NSF-funded project that designs, implements, validates and integrates various interactive tools for statistics and probability education and computing. SOCR resource tools attempt to bridge between the learning and practice of introductory and more advanced computational and applied probability and statistics concepts. The SOCR resource comprises of a hierarchy of portable online interactive aids for motivating, modernizing and improving the teaching format in college-level probability and statistics courses. These tools include a number of applets, user interfaces and demonstrations, which are fully accessible over the Internet [4]–[6]. The SOCR resources allow instructors to supplement methodological course material with hands-on demonstrations, simulations and interactive graphical displays illustrating in a problem-driven manner the presented theoretical and data-analytic concepts. SOCR consists of seven major categories of resources: interactive distribution modeler, virtual experiments, statistical analyses, computer generated games, a data modeler, a data-graphing tool and a newly added java applet on confidence intervals. In this paper, we will demonstrate simulation, construction, validation and interpretation of confidence intervals, under various assumptions, using the interactive web-based tools and materials provided by the Statistics Computational Resource. Specific confidence interval demonstration examples will include intervals for: the population mean, with known or unknown population standard deviation; population variance; population proportion (exact and approximate), as well as confidence intervals based on bootstrapping and using the asymptotic properties of the maximum likelihood estimates. Like all SOCR resources, these confidence interval applets and activities are openly accessible via an Internet-connected computer with a Java-enabled browser [7]. The SOCR confidence interval applet enables the user to empirically explore and investigate the effect of the confidence-level, the sample-size and parameter of interest on the size and location of the corresponding confidence interval. This confidence interval applet also allows random sampling from a large number of discrete and continuous distributions and interactive user-selection of the distribution parameters. These materials are tested and validated in various settings and can be directly integrated in high-school and college curricula.

### Background

Most scientific investigations rely on observable data (quantitative and/or qualitative), which is typically used to develop models, validate diverse research hypotheses, statistically analyze the power of different studies and interpret the intrinsic characteristics of the process of interest. Each observed dataset is assumed to be a representative sample of the population. Throughout this manuscript we are using the common statistics notation denoting by capital letters (e.g., 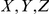) and lower case letters (e.g., 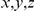) *random variables* and *observed sample values*, respectively. Random samples of 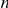 observations may be represented by 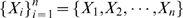. For instance, if 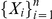 are independent and identically distributed random variables from 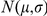 distribution, we can compute interval estimates (ranges) for the population mean using a sample (specific sequence of observations) 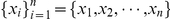. There are several different situations:

1. Depending upon our knowledge of the population variance (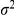), there are two approaches for constructing the confidence interval for the population *mean*, 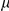.

If the population standard deviation 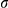 is *known* the confidence interval for the mean 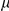 is:
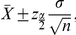
where 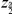 is the 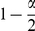 percentile of the 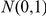 distribution.

If the population standard deviation 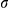 is *unknown*:
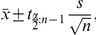
where 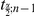 is the 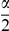 percentile of the student's 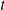 distribution with 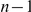 degrees of freedom. Note that this interval is generally wider than the first one.

2. The confidence interval for the population *variance* is obtained by
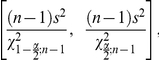
where 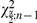 is the 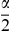 percentile of the 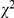 distribution with 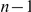 degrees of freedom.

3. Confidence intervals for the population *proportion* may also be constructed in several different ways. Suppose 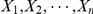 are binary (dichotomous) observations (e.g., “yes” or “no” responses). A simple confidence interval for the population proportion (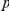) of yes responses is most commonly constructed using the Wald method:
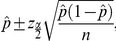
where 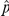 is the sample proportion. However, the Wald confidence interval has poor coverage of the true parameter (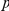) when the sample size is small or when the sample proportion 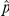 is near zero or near 1. A better estimation of the confidence interval for 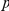 is obtained by:
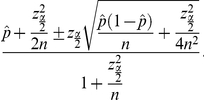



And the best (exact) confidence interval for 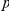, also called the “Clopper-Pearson” interval [8] is computed by:
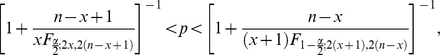
where 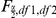 is the 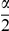 percentile of the 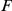 distribution with numerator degrees of freedom 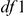 and denominator degrees of freedom 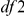.

## Results

**Figure 2:**
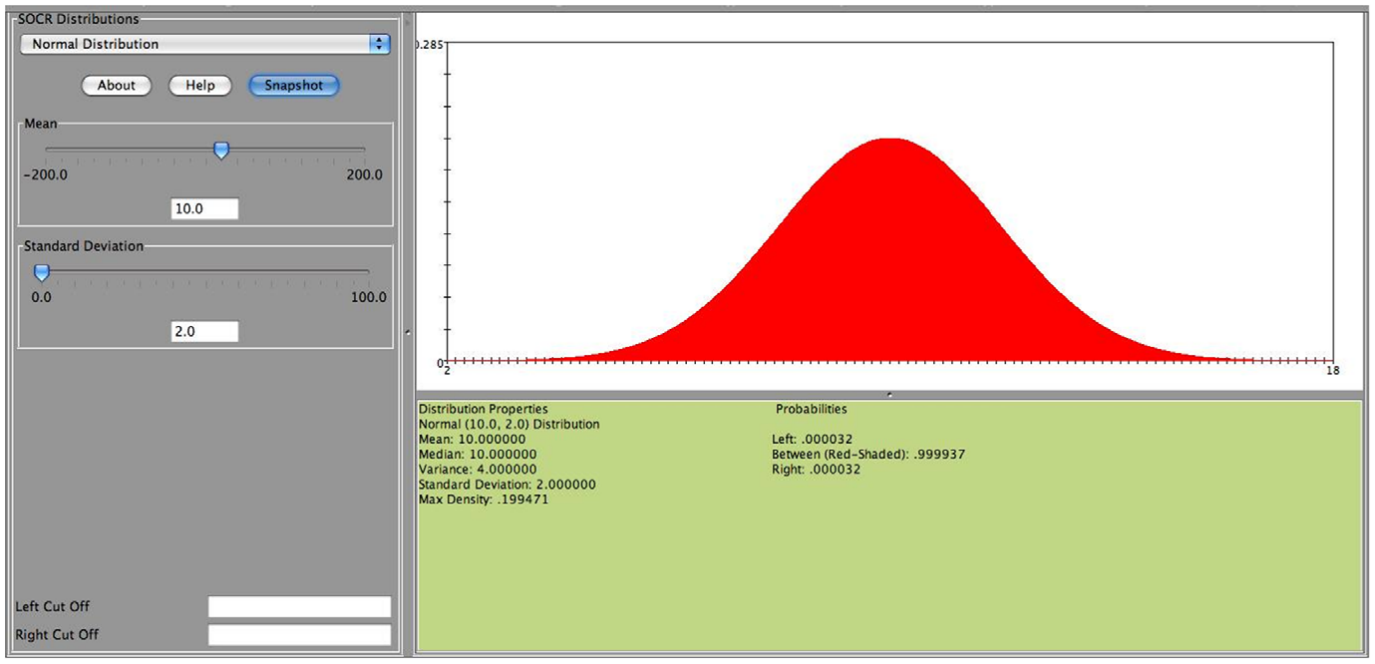
The SOCR distributions applet provides interactive calculation of critical and probability values for over 70 different probability distributions.

The SOCR confidence interval applet is unique in a way that it allows the user to interactively sample from any of the 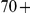 distributions of SOCR (http://www.socr.ucla.edu/htmls/SOCR_Distributions.html) (Figure 2), set the specific parameters of the distribution, select the appropriate confidence interval parameter (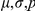, etc.) and then choose the

a. Sample size,

b. Confidence level,

c. Number of intervals to construct.

**Figure 3:**
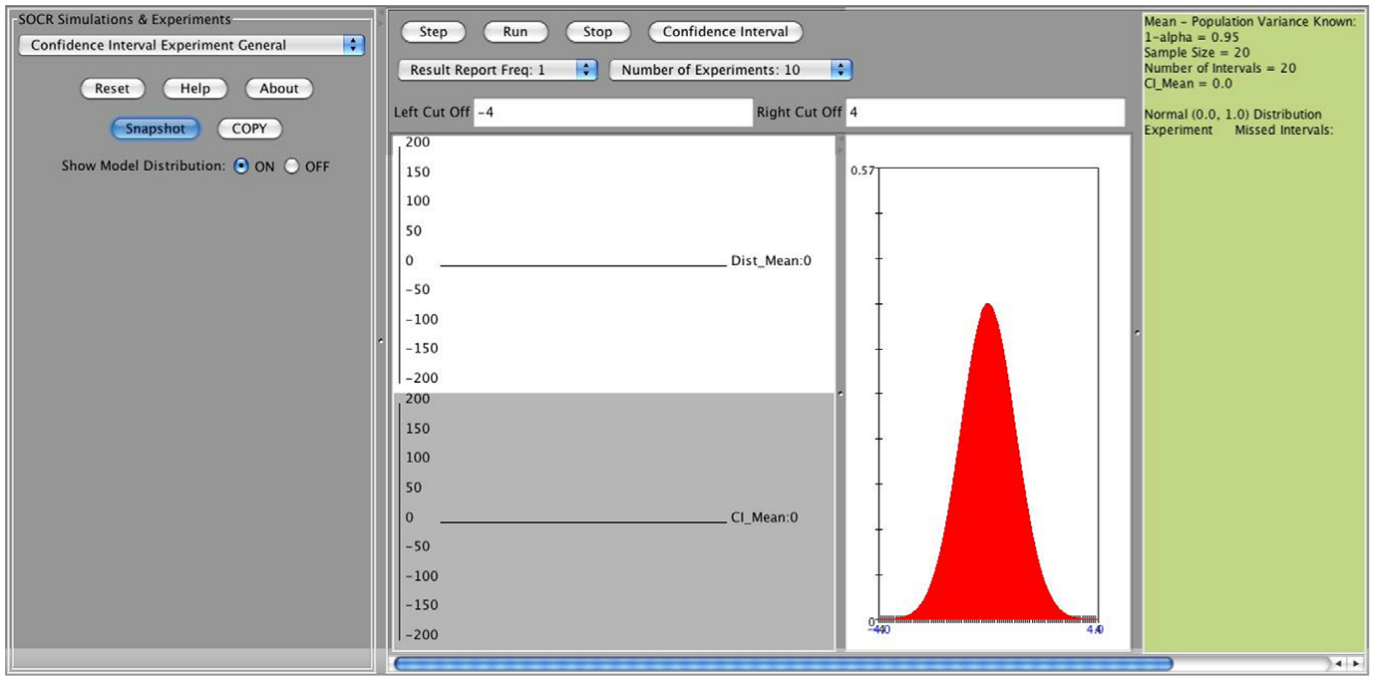
Output window of the SOCR confidence interval applet.

The SOCR confidence interval applet can be accessed directly at (http://socr.ucla.edu/htmls/exp/Confidence_Interval_Experiment_General.html) or via the main SOCR Experiments (http://www.socr.ucla.edu/htmls/SOCR_Experiments.html), select “Confidence Interval Experiment General” from the drop-down menu). The output window of the applet is shown on Figure 3.

**Figure 4:**
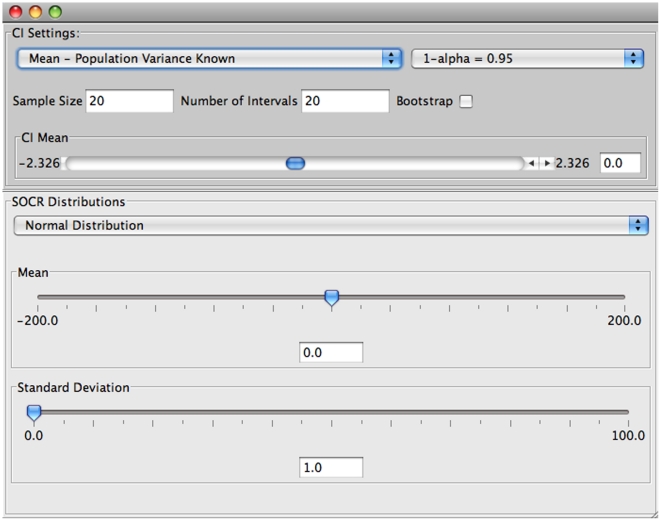
Input window of the SOCR confidence interval applet.

The selection of the various parameters of the experiment can be done through the “Confidence Interval” tab of the main window. Figure 4 shows the default settings of the input window.

In this input window the user can choose:

a. The type of confidence interval to construct (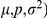.

b. Choose the distribution from where the samples will be taken and enter values for the appropriate parameters of this distribution.

c. Select the sample size.

d. Choose the number of intervals to construct.

e. Set the confidence level (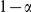).

f. Multiple sample simulation or bootstrapping resampling approach.

**Figure 5:**
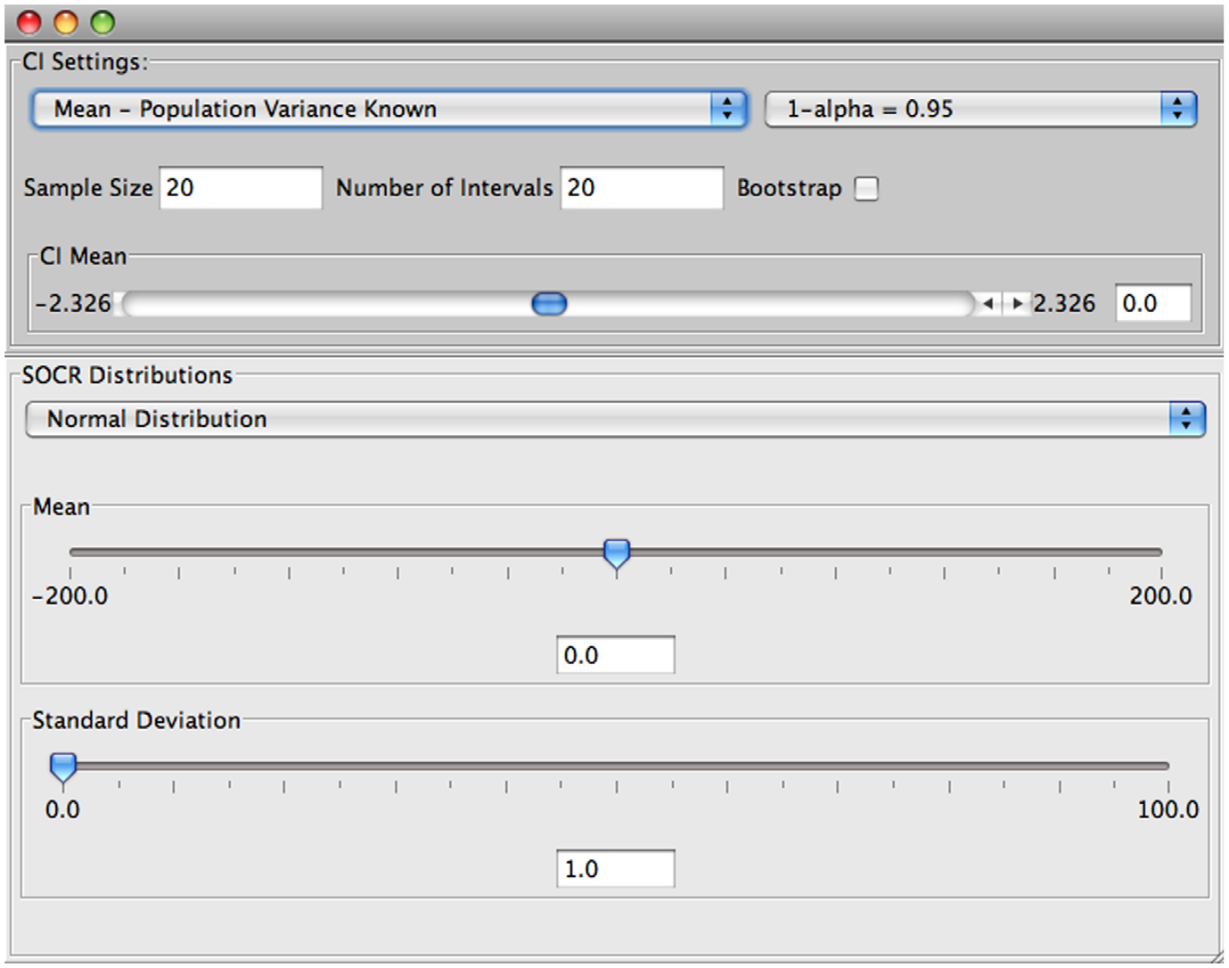
An example: Normal distribution, 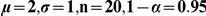, 20 confidence intervals.

Clicking on the “Step” tab on the main applet window will show the results of a single run of the confidence interval (CI) experiment, using the user specified parameters, Figure 5.

**Figure 6:**
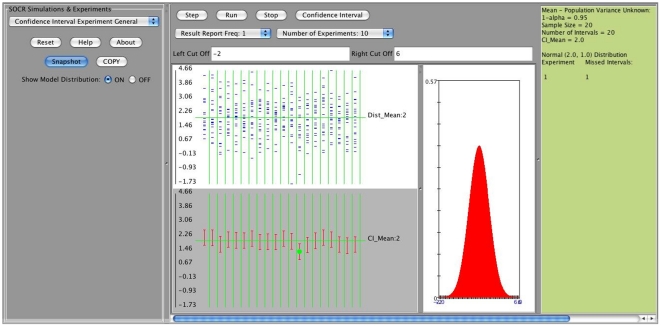
Results of a single run of the CI experiment.

**Figure 7:**
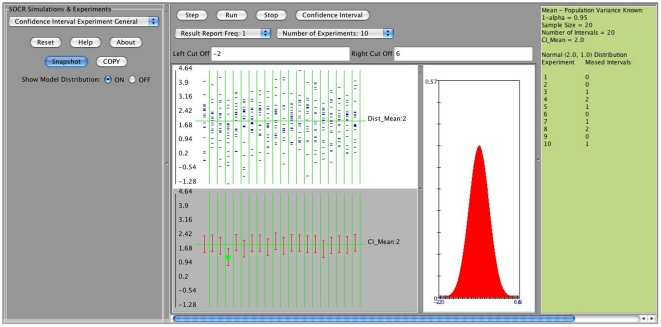
Results of 10 runs of the experiment.

We observe that all the input parameters are recorded on the right margin of the applet's main window (Figure 6). There are two displays on this window. The top display shows the distribution mean and the sample values (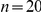 in our example) selected for each interval (we have constructed 20 intervals). The second display depicts the actual 20 intervals which are constructed from the 20 random samples (red segments). When a confidence interval misses the true mean (here 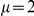) a green dot is shown to indicate this discrepancy. Observe that in this example because we have assumed that the population standard deviation is known (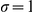) all the intervals have the same length. The confidence level was chosen to be 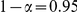 and therefore it is not surprising that among the 20 intervals only one missed the target parameter (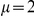). If we choose to run the experiment multiple times (for example 10 times), we simply select the “Number of Experiments  = 10” and then click on the “Run” button. The results from each run of these 10 experiments are recorded on the right margin of the applet and are shown on Figure 7. In general, as these are random simulations, repeats of the experiment using the same parameter settings would generate different outcomes (sample instances and corresponding confidence intervals).

We can now access again the input window and observe how changes of the parameters may affect the appearance (size and location) of the confidence intervals. For example, if we increase the sample size, we will observe narrower confidence intervals. On the other hand, increasing the confidence level will generate higher interval coverage (more intervals will include the actual population mean parameter), however the width of the CIs will increase.

**Figure 8:**
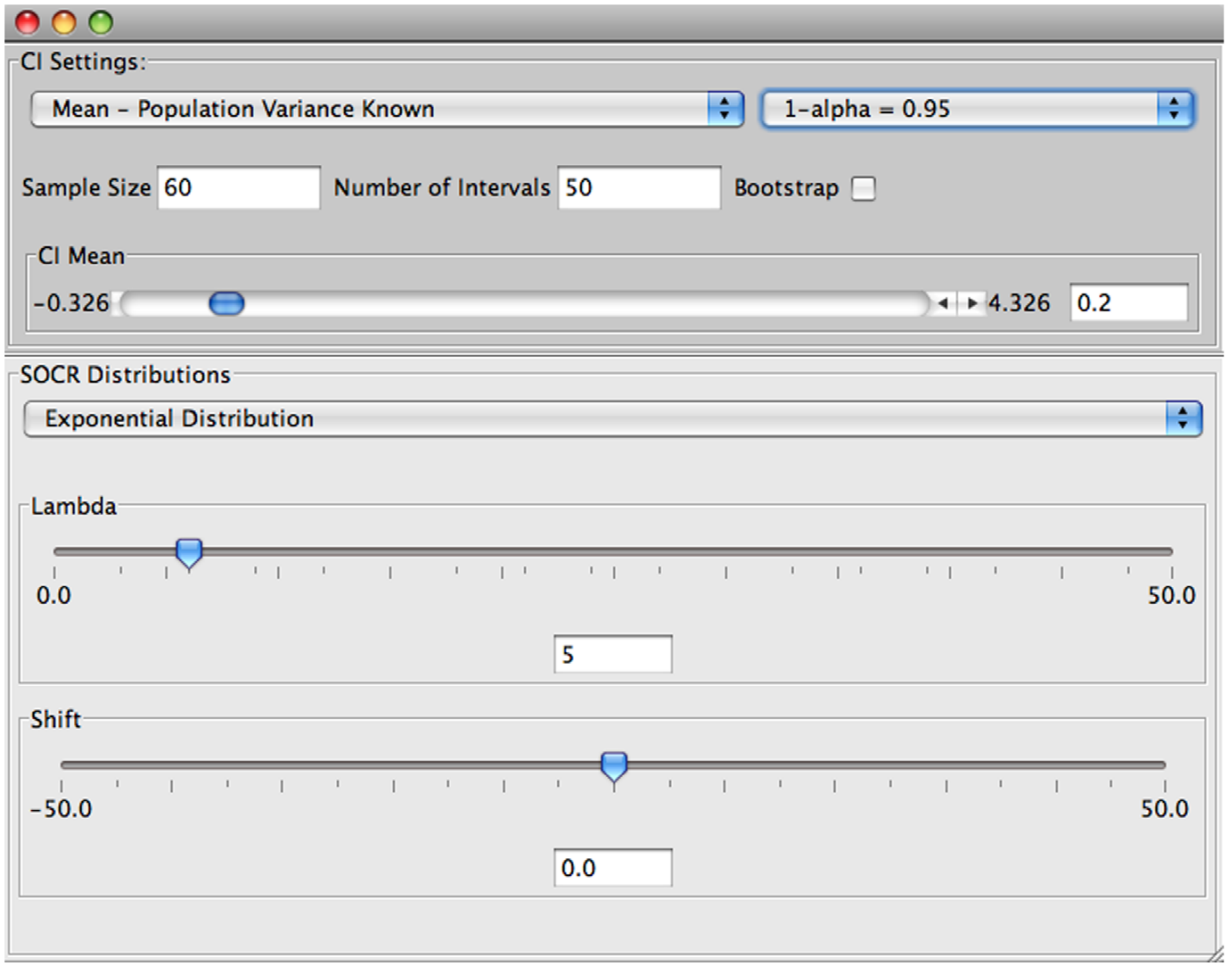
Input of the experiment when 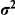 is not known.

**Figure 9:**
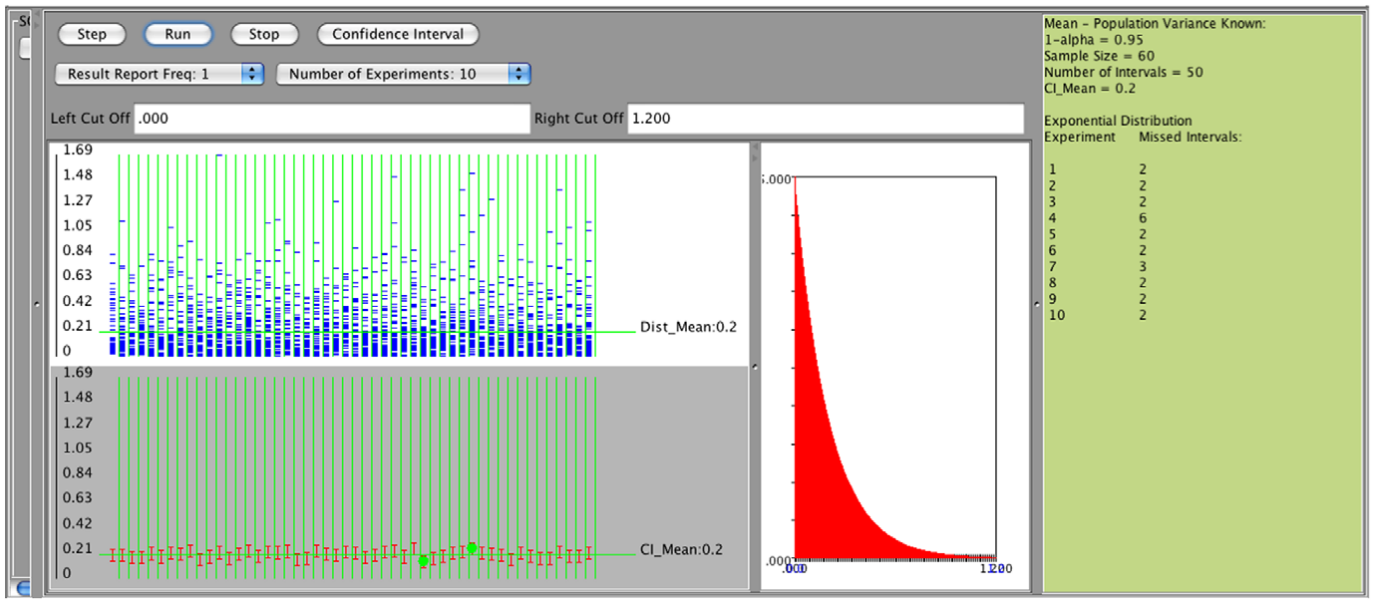
Results of 10 runs of the experiment.

Confidence intervals for the mean with unknown standard deviation can be constructed analogously. These confidence intervals are based on the 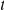 distribution and the length of different intervals will vary because the specific sample standard deviation is used in their construction. Consider the exponential distribution with parameter 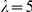 (mean of 0.2), sample size 60, confidence level 0.95, and number of intervals 50. Results of this experiment are shown on Figure 8 and Figure 9.

**Figure 10:**
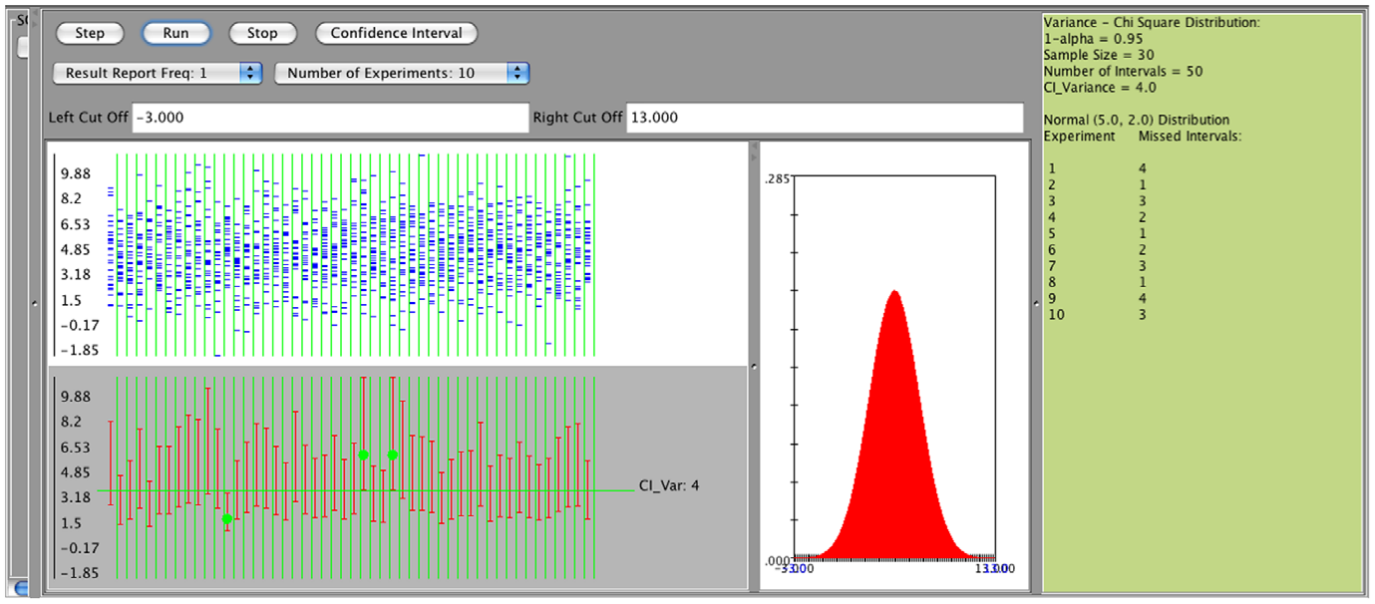
Confidence intervals for 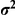 - sampling from normal distribution.

We now examine the confidence interval for the population variance 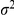. Suppose we first use the confidence intervals applet to sample from a normal distribution with mean 5 and standard deviation 2, specifying sample size 30, confidence intervals 50, and confidence level 0.95. We observe that the coverage is indeed about 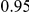 (Figure 10 - see results on the right margin of the applet). Again, this is not surprising since when sampling from normal distribution the confidence interval is based on the chi-square distribution.

**Figure 11:**
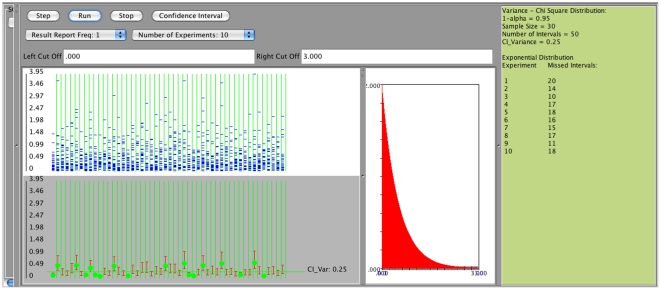
Confidence intervals for 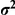 - sampling from non-normal distribution, e.g. exponential, 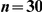.

**Figure 12:**
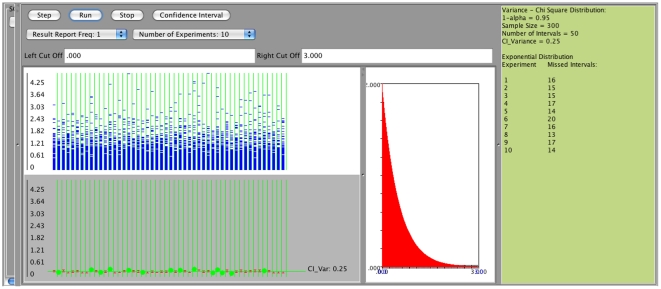
Confidence intervals for 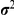 - sampling from non-normal distribution, 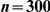.



**Figure 13:**
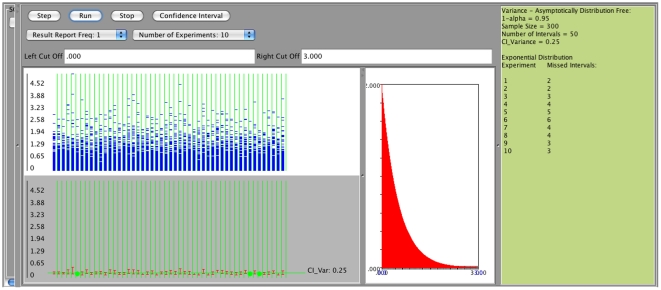
Large samples confidence intervals for 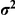 - sampling from non-normal distribution.

However, if the population is not normal the interval coverage is poor as can be seen in the following SOCR example. Consider the exponential distribution with 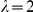 (variance is 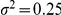). If we use the confidence interval based on the 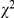 distribution we obtain the following results (first with sample size 30 and then sample size 300), Figure 11. Specifically, we observe that in both cases (regardless of the sample size - small or large) the coverage is poor, significantly less than 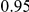. For example, on Figure 11, a meta-experiment, each consisting of generating 50 intervals of sample size 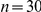, yields an overall average number of confidence intervals that fail to cover the true population variance equals to 16. On Figure 12 we see the same poor coverage even when the sample size increases to (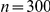). In these situations (sampling from non-normal populations), an asymptotic distribution-free confidence interval for the variance can be obtained using the following large sample theory result [9]:

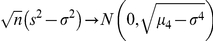
or,
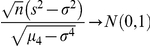
where, 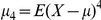 is the fourth moment of the distribution. Of course, 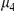 is unknown and will be estimated by the fourth sample moment 
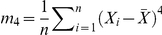
. The confidence interval for the population variance is then computed as follows:
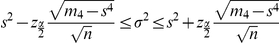



Using the SOCR confidence intervals applet (exponential distribution with 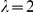, sample size 300, number of intervals 50, confidence level 0.95), we observe an approximate interval coverage of 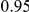, Figure 13.

**Figure 14:**
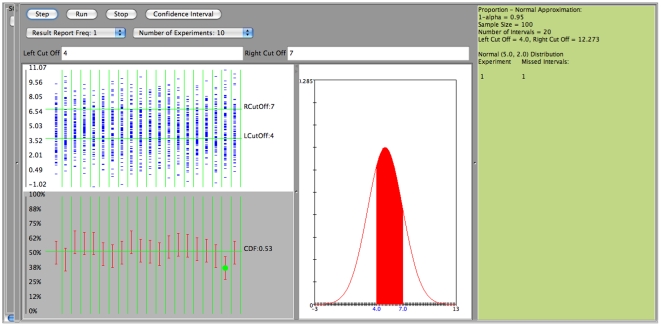
Confidence interval for proportion 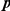: Sampling from 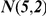.

We will examine now the confidence interval for the population parameter 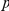. The SOCR confidence interval applet provides calculation for the three types of confidence interval for 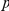 mentioned in the Background Section. The applet's unique feature is that the user can again sample from any of the available SOCR distributions and define what the meaning of a success is. Success is defined as *a randomly chosen observation falling within a user-specified interval*. Success intervals are selected by drawing left and right interval limit on the distribution curve using the mouse, or by entering appropriate numerical limits in the distribution left and right text fields (see Figure 14). For example, suppose we choose to sample 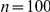 observations from a normal distribution with mean 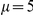, and standard deviation 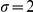. The user may define as success any observations that fall between 4 and 7 or any other valid range. Once this is done, it is easy to compute the sample proportion using 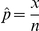 where 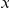 is the number of observations that fall between 4 and 7, and 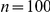.

**Figure 15:**
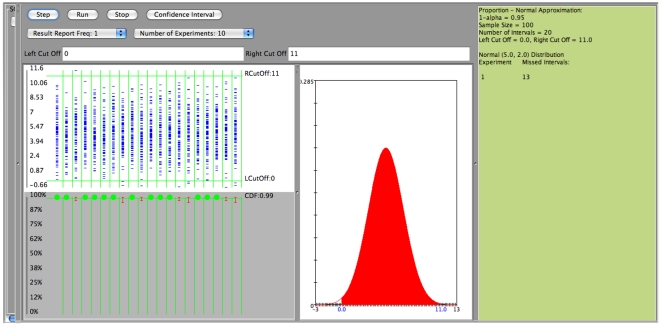
Confidence interval for proportion 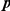: Poor coverage using the Wald confidence interval.

**Figure 16:**
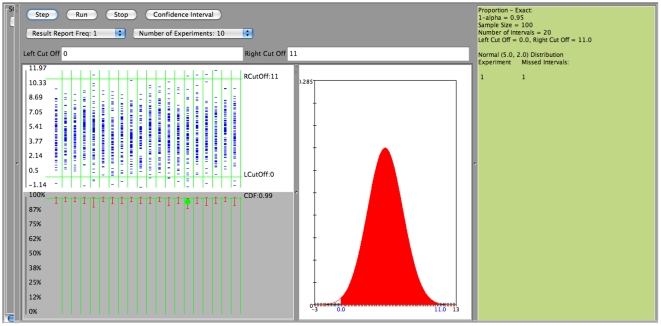
Confidence interval for proportion 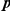: Good coverage using the Clopper-Pearson confidence interval.

In the next two figures we are presenting the case of poor coverage of the Wald confidence interval (Figure 15 when 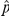 is large (close to one), and the much better coverage for the same case using the Clopper-Pearson (exact) confidence interval (Figure 16). In both cases we sample 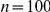 observations from 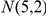, a 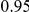 confidence level is used, and success is defined by an observation falling between 0 and 11.

In addition, the SOCR confidence interval applet provides interval estimation for population parameters of a distribution based on the asymptotic properties of maximum likelihood estimates. This is based on the large sample theory result of maximum likelihood estimates.

As the sample size 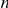 increases it can be shown [10] that the maximum likelihood estimate (MLE) 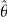 of a parameter 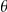 follows approximately normal distribution with mean 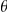 and variance equal to the lower bound of the Cramer-Rao inequality [10].
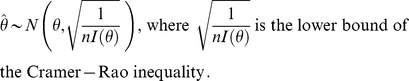



Because 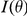 (Fisher's information) is a function of the unknown parameter 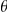, the parameter is replaced by its maximum likelihood estimate 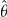 to get 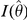.

Since,
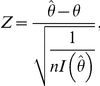
we can write
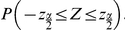



We replace 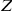 with 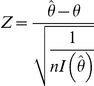 to get
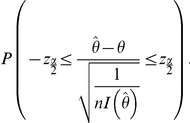



And finally,
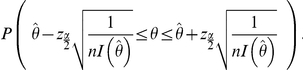



Therefore, we are 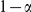 confident that 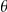 falls in the interval
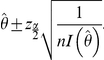



This result is used in the example below to construct a confidence interval for Poisson distribution with parameter 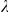. Let 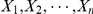 be independent and identically distributed random variables from a Poisson distribution with parameter 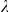. We know that the maximum likelihood estimate of 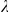 is 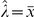. We need to find the lower bound of the Cramer-Rao inequality:
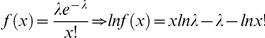



Let's find the first and second derivativeswith respect to 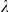.
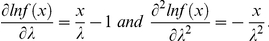



Therefore,
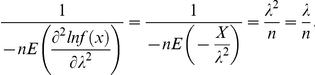



When 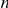 is large, 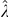 follows approximately
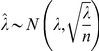



Because 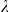 is unknown we replace it with its MLE estimate 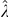:
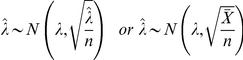



Hence, the confidence interval for 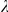 is:
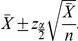



Studies of the number of pine trees in certain forests provide interesting applications. Suppose the number of pine trees per acre follows Poisson distribution with unknown parameter 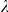. If we select a random sample of size 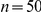 acres and count the number of pine trees in each acre. The following sample of observations represents the real measurements for the number of trees in 50 one-acre areas:

7 4 5 3 1 5 7 6 4 3 2 6 6 9 2 3 3 7 2 5 5 4 4 8 8 7 2 6 3 5 0 5 8 9 3 4 5 4 6 1 0 5 4 6 3 6 9 5 7 6.

The sample mean is 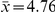. Therefore a 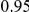 confidence interval for the parameter 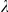 would be
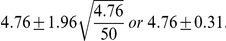



In other words, 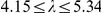.

**Figure 17:**
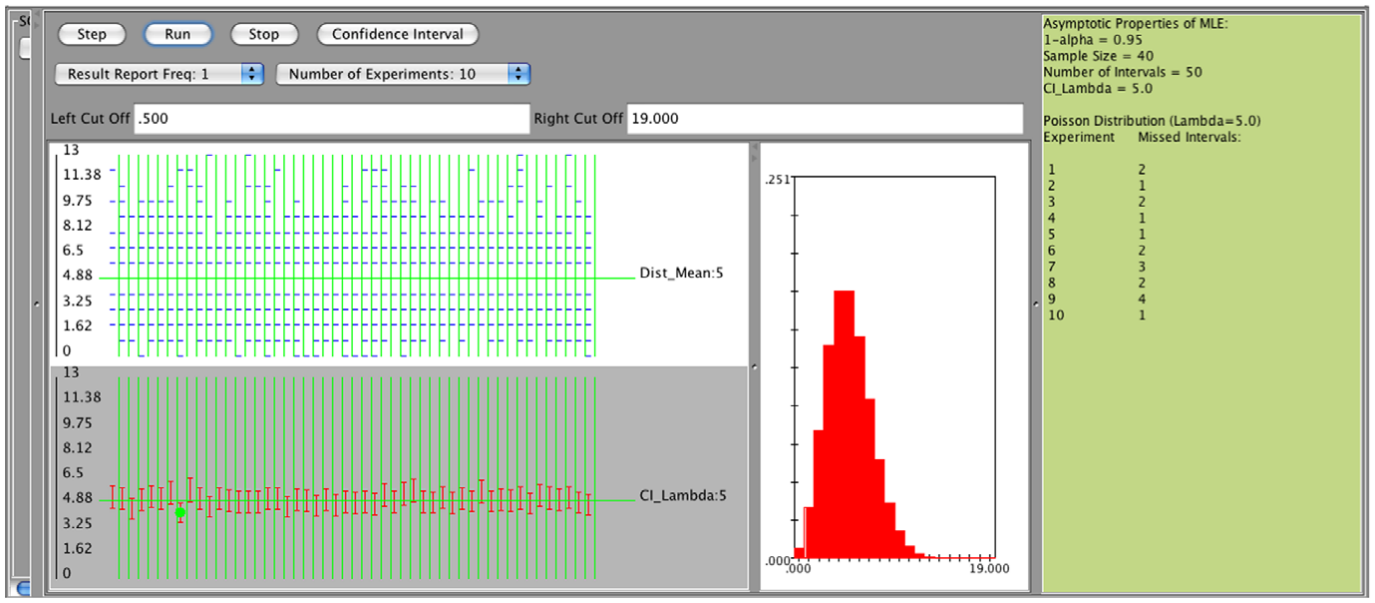
Confidence intervals using asymptotic properties of maximum likelihood estimates - Poisson distribution with parameter 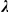.

**Figure 18:**
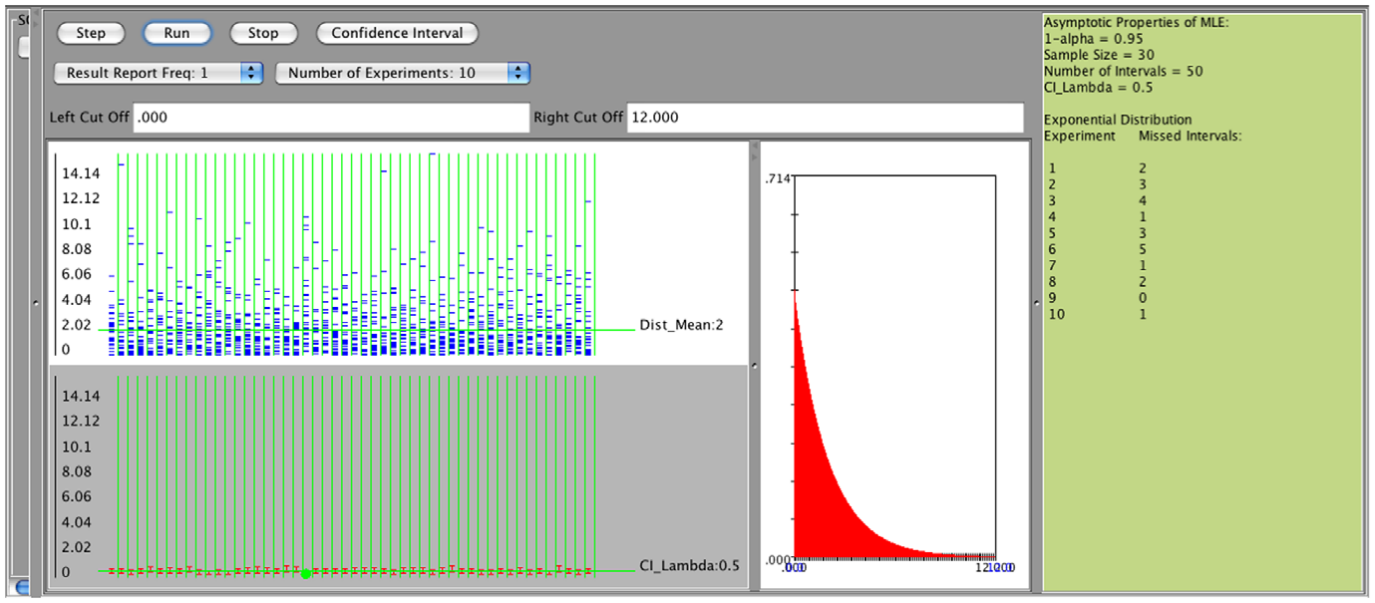
Confidence intervals using asymptotic properties of maximum likelihood estimates - Exponential distribution with parameter 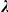.

Using the same method, a confidence interval for the parameter 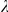 of the exponential distribution may be obtained. It can be shown that the confidence interval obtained by this method is given as follows:
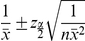



Currently the SOCR confidence interval applet provides these two intervals using the asymptotic properties of maximum likelihood estimates. The following SOCR simulations (Figure 17 and Figure 18) refer to

a. Poisson distribution, 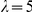, sample size 40, number of intervals 50, confidence level 0.95, Figure 17.

b. Exponential distribution, 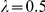, sample size 30, number of intervals 50, confidence level 0.95, Figure 18.

The following two applications of the new SOCR Confidence Interval applet demonstrate the practical usage of these new statistical computing resources.

**Figure 19:**
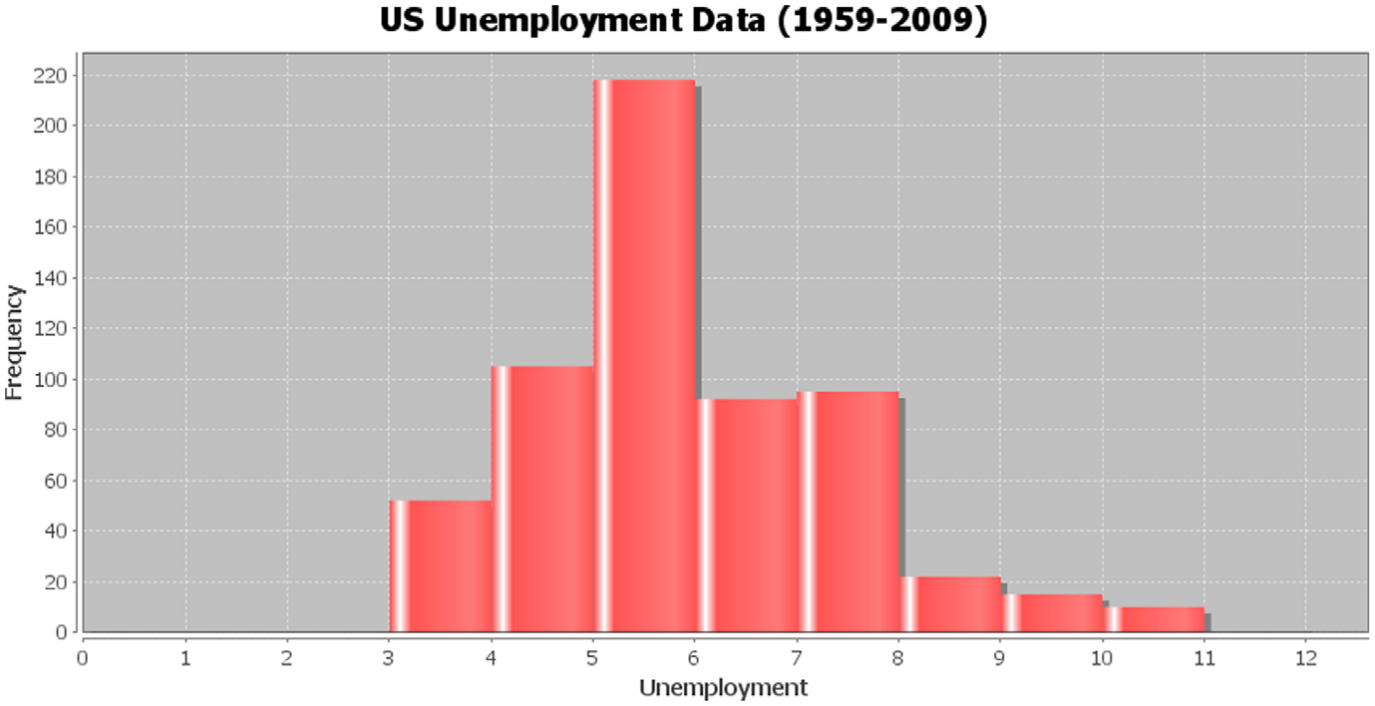
US Unemployment distribution (1959–2009).

The first application shows a study of US unemployment. One question is how can we find the 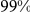 confidence interval for the proportion (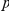) of (officially) unemployed workers in the US. We begin by looking at some of real unemployment data for the period 1959–2009 (http://wiki.stat.ucla.edu/socr/index.php/021111) provided by the Federal Reserve Bank of St. Louis, MO. These data contain a number of economic indicators for a 50-year time span. The distribution of unemployment is shown on Figure 19.

**Figure 20:**
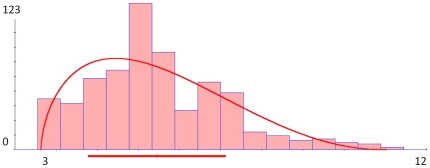
Modeling the unemployment data using generalized Beta distribution. The coordinate axes represent X  =  (values) unemployment rate and Y  =  (frequencies) number of months (1959–2009) when unemployment rate was at the given X value.

Next, we fit in a generalized Beta distribution model to the frequency distribution of the unemployment data. Figure 20 shows the Beta model fit density curve juxtaposed on top of the unemployment data distribution. The maximum likelihood estimates of the four Beta distribution parameters are obtained using the SOCR Modeler (http://socr.ucla.edu/htmls/SOCR_Modeler.html):

Left (Shape) Parameter (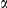)  =  1.5903

Right (Shape) Parameter (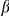)  =  3.1453

Left (Location) Limit  =  3.4

Right (Location) Limit  =  10.8

Kolmogorov-Smirnoff (KS) test was used to determine that the differences between the unemployment data distribution and Beta model distribution are not statistically significant (KS D-Statistics  =  0.07347, Z-Statistics  =  0.51948, P-value  =  0.921).

**Figure 21:**
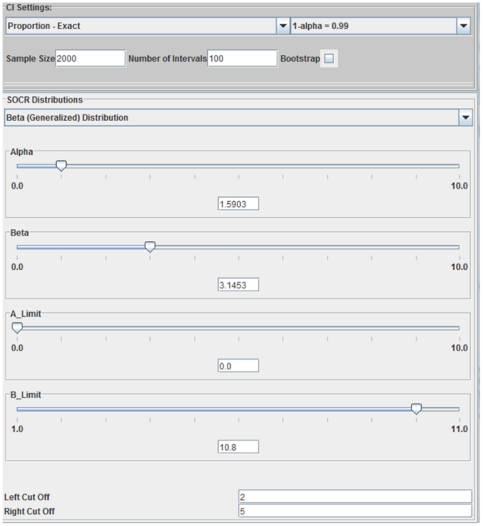
SOCR Confidence interval simulation settings panel for estimating the unemployment rate (proportion of US unemployed workers) using the generalized Beta (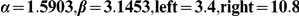) distribution model described above.

**Figure 22:**
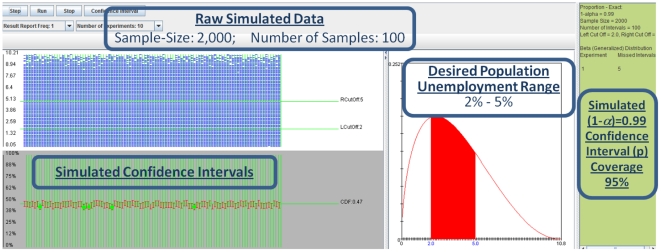
Results of 100 simulations (samples include N = 2,000 random observations from Beta distribution) of 0.99 confidence intervals for the population proportion (using the exact method) provide effective coverage of 95%. Five of the 100 simulations miss the real population proportion *p* = 0.47, which represents the shaded area below the Beta density function. This proportion indicates a healthy US unemployment rate between 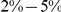.

Then, we can run a simulation and estimate the confidence interval for the proportion of unemployment population to be within the desirable range of 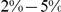 (note that 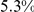 is considered “full employment”). Figure 21 shows the 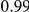 confidence interval simulation settings panel, and Figure 22 illustrates the results of the simulation. The 100 simulations of 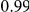 confidence intervals for the population proportion use the exact method and provide effective coverage of 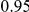. In other words, 5 of the 100 simulations miss the real population proportion (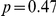 representing the shaded area below the Beta density function on Figure 22, which indicates a healthy unemployment rate between 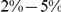). Note that each of the 100 samples contains 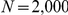 random observations from Beta distribution. The discrepancy between the expected 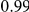 coverage and the observed 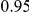 coverage rates for the simulated confidence intervals of the population proportion can be explained by random sampling variation or the small number of intervals (100). A larger number of simulations using 1,000 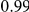 confidence intervals cover the population proportion of interest (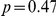) 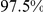 of the time (or 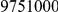).

Finally, we can use the analysis portion of the SOCR confidence interval analysis applet (http://socr.ucla.edu/htmls/ana/ConfidenceInterval_Analysis.html) to estimate the 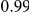 confidence intervals for the expected (mean) unemployment rate in the US using the available sample data of 609 monthly unemployment measurement for 1959–2009: [5.732, 6.038], with a 50-year unemployment median of 5.885.

**Figure 23:**
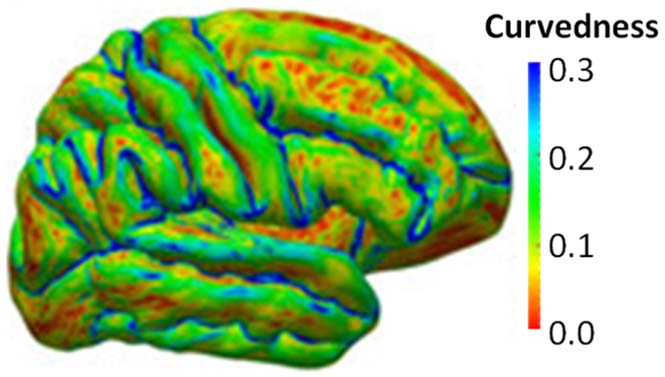
Illustration of the relation between local cortical folding patterns and the values of the curvedness measure computed for each vertex on the shape. Averaging all local curvedness measures over the entire surface provides a global curvedness index measuring the overall complexity of a shape. This figure shows the left lateral view of the cortical surface of one subject color coded by the local curvedness.

The second application of the SOCR confidence interval computational library involves a large neuroimaging study where automated volumetric data processing [11] is used to obtain different shape and volume measures of local brain anatomy. The subject population is derived from the Alzheimer's Disease Neuroimaging Initiative (ADNI) database (http://ADNI.loni.ucla.edu/) [12] and includes 27 Alzheimer's disease (AD) subjects, 35 normal controls (NC), and 42 mild cognitive impairment subjects (MCI). The broad goal of this study is to identify associations and relationships between neuroimaging biomarkers and various subject demographics and traits. For instance, we use the Bootstrapping method to construct 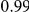 confidence intervals for the mean curvedness (measure of shape) for the left and right hippocampus for each of the three cohorts. Given the two principal curvatures 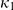 and 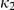 [13], where 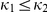, the curvedness (CV) is defined by:
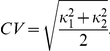



The CV shape measure is computed at each vertex on the shape and we used the global curvedness, which is the overall average of local curvedness measured at each hippocampal surface vertex. Figure 23 shows an example of the local curvedness map on the left hemisphere of the cortical surface for one subject.

This neuroimaging dataset is available online (http://wiki.stat.ucla.edu/socr/index.php/021211). It contains (deidentified) subject index, group indicator (AD, NC, MCI), two cognitive assessment measures: MMSE (Mini-Mental State Exam) score and CDR (Clinical Dementia Rating) scale, subject's gender, age, TBV (Total Brain Volume) measure, total GMV (Gray Matter Volume), total WMV (White Matter Volume), total CSFV (Cerebrospinal Fluid Volume), and a numerical value for four shape-based measures (Surface Area, Shape Index, Curvedness and Fractal Dimension) for each of 56 brain Regions of Interest (ROIs) [14]. All of these measures are extracted using the Global Shape Analysis protocol via the LONI Pipeline environment [11]. Here we only demonstrate the Bootstrap based construction of the confidence intervals for the average left and right hippocampal *curvedness measure* for each of the three cohorts, Table 1. These results clearly show a monotonic increase of the curvedness shape measure between the 3 cohorts. This trend indicating group differences of the centers (medians) and dispersion (width) of estimated confidence intervals supports prior studies indicating progressive hippocampal anatomical atrophy reported in dementia subjects as they progress from NC to MCI and AD [15]–[18].

**Table 1:** Bilateral (left and right) point (median) and interval (0.99 confidence intervals using bootstrapping with 20,000 resampling simulations) estimates for the hippocampal surface complexity (measured using curvedness) for the 3 cohorts.

Cohorts	Left Hippocampus		Right Hippocampus	
	Curvedness Shape Measure		Curvedness Shape Measure	
	Median	0.99 Confidence Interval	Median	0.99 Confidence Interval
Alzheimer's				
Disease	0.2081	[0.1989, 0.2174]	0.2044	[0.1944, 0.2152]
(*N* = 27)				
Mild				
Cognitive	0.1971	[0.1924, 0.2017]	0.1952	[0.1886, 0.2021]
Impairment				
(*N* = 42)				
Normal				
Controls	0.1974	[0.1906, 0.2043]	0.1857	[0.1789, 0.1929]
(*N* = 35)				

Similarly, for the same dataset we may obtain point and interval estimates for other parameters of interest (e.g., variance, various types of proportions) for specific ROI and shape measure based on any of the available confidence interval methods included in the SOCR CI computational library.

## Discussion

Interval estimation of population parameters is an important component of many quantitative scientific investigations. Algorithmic constructions of interval estimates depend on a number of different factors, e.g., the characteristics of the natural distribution of the process, parameter of interest, computational efficiency and stability of the estimates, and sample size. In addition, there are different approaches for obtaining parameter interval estimates. For instance, there are completely automated and semi-automated techniques for constructing confidence intervals facilitating quantitative statistical analysis. Automatic protocols for obtaining confidence intervals (CIs) represent software programs which compute the intervals directly (using the data and the process density function). Some of the examples above show that in certain situations the interpretations of these intervals may significantly deviate from our common understanding of confidence intervals. Variable transformations (e.g., 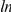, 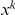, 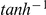) are known to help aligning the theoretical approaches, algorithmic implementations and the practical interpretations of confidence intervals. However, the choice of an appropriate transformation is mostly subjective and requires input from investigators. There are also completely automated approaches to produce *approximate* confidence intervals. Confidence intervals bootstrapping is one notable example [19], [20], which requires no special expert intervention. Drawbacks of such techniques include significant dependence of the interval on small variations in the data and the large amount of computing required to obtain the interval estimates (often 10,000Õs of iterations of bootstrap CI calculation are required) [21]. Completely automated nonparametric CI estimates are always approximate since exact estimates do not exist for most parameters [22].

There are a number of pedagogical challenges in motivation, application and interpretation of confidence intervals for different population parameters [23]. Some of these challenges relate to the underlying assumptions, applications or limitations, accuracy and validity of the confidence intervals, as well as the interplays between sample size, confidence level and process density function. Graphical renderings and interactive simulations of data sampling and CI calculations may require some technical expertise and programming skills, but provide powerful instructional aides for training novice researchers and junior investigators. A direct example of these pedagogical challenges is the interrelation between the desirable narrow-width confidence interval and the expectation for high level of confidence.

Theory, construction and interpretation of CIÕs may all present problems for learners and practitioners of quantitative statistical methodologies. Frequent refreshers and reinforcement of these ideas using interactive graphical tools improves the knowledge retention and understanding the role of the *sample* in the construction of dynamic confidence intervals with each repetition of the experiment.

Computational challenges present another barrier for many confidence intervals learners, applied scientists and clinical investigators [24], [25]. There are two specific types of CI computational challenges. The first one is identifying the distribution of the parameter of interest and developing a computationally-tractable algorithmic approach for estimating the parameter (and its moments). The second one is the implementation of (accessible) software tools that provide efficient and robust numerical CI estimates for datasets with varying characteristics (e.g., scale, heterogeneous format, sample-size).

The SOCR confidence intervals applet allows simulation and validation of various protocols for interval estimation using random simulations. In practice, most investigators and learners need to construct interval estimates for various parameters of interest using data obtained via research or observational protocols. The open-source SOCR library enables the integration of these calculations as part of any web-based or stand-alone computational tool. Complete Java documentations of this library is available here (http://www.socr.ucla.edu/docs). An instance demonstrating the usage of this CI computational library is included in the SOCR Analysis package [5]. The interactive SOCR confidence intervals analysis applet (http://www.socr.ucla.edu/htmls/ana/ConfidenceInterval_Analysis.html) enables the user to enter any (numerical) data, specify a parameter of interest, select an interval generation protocol, and compute the corresponding data-driven interval estimate. The applet provides several default datasets, however researchers can load or paste in external tabular data in the applet's data tab. The source code for this CI analysis applet demonstrates the externals invocation protocol of the SOCR CI calculations and is also freely available online.

This manuscript presents a unified, open-source, portable and extensible computational framework for computing, simulating and visualizing confidence intervals estimates in a broad spectrum of conditions. These resources address many of the common instructional, computational and application challenges described above. We showed two applications of the new interval estimation computational library. The first one is a simulation of confidence interval estimates for the proportion of years when a healthy 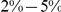 unemployment rate may be expected and an estimation of the confidence interval for the US unemployment rate. The second application demonstrates the computations of point and interval estimates of hippocampal surface complexity for three cohorts. The source code, web-applet and interactive learning activity are all freely and anonymously accessible online (http://wiki.stat.ucla.edu/socr/index.php/092110).
